# Alpha-1-antitrypsin phenotypes in adult liver disease patients

**DOI:** 10.3109/03009730903243472

**Published:** 2009-12-08

**Authors:** Aleksandra Topic, Tamara Alempijevic, Aleksandra Sokic Milutinovic, Nada Kovacevic

**Affiliations:** ^1^Institute of Medical Biochemistry, Faculty of Pharmacy, University of Belgrade, BelgradeSerbia; ^2^Clinic of Gastroenterohepatology, Institute for Digestive Diseases, Clinical Center of Serbia, BelgradeSerbia

**Keywords:** Adults, alpha-1-antitrypsin, liver disease, phenotypes

## Abstract

Alpha-1-antitrypsin (AAT) is an important serine protease inhibitor in humans. Hereditary alpha-1-antitrypsin deficiency (AATD) affects lungs and liver. Liver disease caused by AATD in paediatric patients has been previously well documented. However, the association of liver disease with alpha-1-antitrypsin gene polymorphisms in adults is less clear. Therefore, we aimed to study AAT polymorphisms in adults with liver disease. We performed a case-control study. AAT polymorphisms were investigated by isoelectric focusing in 61 patients with liver cirrhosis and 9 patients with hepatocellular carcinoma. The control group consisted of 218 healthy blood donors. A significant deviation of observed and expected frequency of AAT phenotypes from Hardy-Weinberg equilibrium (chi-square = 34.77, df 11, *P* = 0.000) in the patient group was caused by a higher than expected frequency of Pi ZZ homozygotes (*f* = 0.0143 and *f* = 0.0005, respectively, *P* = 0.000). In addition, Pi M homozygotes were more frequent in patients than in controls (63% and 46%, respectively, *P* = 0.025). Our study results show that Pi ZZ homozygosity in adults could be associated with severe liver disease. Presence of Pi M homozygosity could be associated with liver disease via some mechanism different from Z allele-induced liver damage through accumulation of AAT polymers.

## Introduction

Alpha-1-antitrypsin (AAT) is a glycoprotein also referred to as SERPINA1 (SER*ine* P*roteinase* In*hibitors*, clade A, member 1), since it is a member of serine protease inhibitor (serpin) family. The AAT molecule is produced mainly in the liver and, in smaller quantities, in macrophages and in the bronchial epithelium. Through the circulation, AAT reaches the lungs, where it will perform its anti-elastolytic function. AAT is also an acute phase protein. AAT is encoded in the *SERPINA1* gene, and more than 100 alleles have been identified to date. Approximately 30 of these alleles may have clinical implications. Based on serum levels of AAT and molecular function, the Pi (P*rotese* i*nhibitor*) variants are classified into four groups: normal (M alleles), deficient (serum AAT less than one-sixth of normal values, Z alleles), dysfunctional (normal serum AAT but with reduced function, such as F and Pittsburgh alleles) and null (undetectable serum level of AAT, QO alleles). Deficient, dysfunctional and null variants are related to clinical disease.

AAT serpinopathies (serpin-related diseases) can affect lung and liver (1). Molecular pathology is complex and includes intracellular accumulation of mutant AAT, increased activity of proteolytic cascade, and tissue damage due to the AAT extracellular deposits.

Among all the variants related to clinical disease, mutations Z and S are the most common and are a consequence of point mutations in the AAT gene.

The Z mutation (Glu^342^→Lys^342^) is present in 4% of North Europeans and results in a severe decrease in plasma AAT. In Z mutation homozygotes (Pi ZZ), plasma AAT levels are decreased to 10%–15% of those found in the normal M allele-carrying individuals. In Z mutation carriers posttranslational modification of AAT synthesis occurs, leading to accumulation of polymers in the cisterna of the rough endoplasmic reticulum and a reduction of AAT secretion. Aberrant AAT protein accumulated in the rough endoplasmic reticulum of the liver is seen as periodic acid-Schiff-positive (PAS-positive), diastase-resistant inclusions. Accumulated AAT aggregates in alpha-1-antitrypsin deficiency (AATD) cause hepatocyte death and liver disease, including juvenile liver cirrhosis and development of hepatocellular car cinoma (2).

The S mutation (Glu^264^→Val^264^) found in up to 28% of South Europeans is not associated with clinical disease, although AAT plasma levels are 60% of those found in M allele carriers. In contrast to the Z mutation, the S allele causes only mild plasma deficiency due to reduced secretion of AAT. In these individuals newly synthesized AAT is degraded in the cell prior to secretion (3,4). Nevertheless, individuals who are SZ heterozygous (Pi SZ) have liver disease (5).

AATD is a genetic disorder that besides the above-mentioned liver disease leads to lung injury and early development of emphysema in adults and accounts for 1%–3% of all cases of chronic obstructive pulmonary disease (COPD) (6,7).

AATD in adults manifests as COPD in 60% of all patients, while only 12% of adult patients have liver cirrhosis. The simultaneous presence of COPD and liver cirrhosis is rare.

The aim of our study was to determine the frequency of AAT mutations in adults with severe liver disease and assess if differences in liver function tests related to the presence of Z allele exist.

## Material and methods

We included 70 patients in the study group: 61 treated for liver cirrhosis and 9 diagnosed with hepatocellular carcinoma (HCC) at the Clinic for Gastroenterology and Hepatology, Clinical Centre of Serbia. Diagnosis was based on clinical features, laboratory tests, imaging diagnostics, and liver histology whenever possible. The Ethics Committee approved the study, and all patients gave an informed consent prior to inclusion into this investigation.

The following data were registered for each patient: age, gender, aetiology of cirrhosis, biochemical parameters (aspartate aminotransferase, alanine aminotransferase, total bilirubin, serum albumin, prothrombin activity, and platelet count), presence and degree of oesophageal varices, and degree of liver function impairment by Child-Pugh classes. Cirrhosis aetiology was determined as viral if hepatitis B surface antigen or hepatitis C serum markers were positive. If viral markers were negative, and the history obtained indicated alcohol consumption of at least 50 g per day in the past 5 years, liver cirrhosis was considered as alcoholic. Positive immunological markers were characteristic for immunological liver disease. The other studied cases had liver cirrhosis of different aetiology (Wilson's disease, AATD, haemochromatosis, etc.). Diagnosis of HCC was based on imaging techniques (ultrasonography, computed tomography, and NMR), as well as elevated alpha-fetoprotein (AFP) and liver histology.

The Child-Pugh score was calculated using five variables (ascites, encephalopathy, bilirubin, albumin, and prothrombin time). Values of 1, 2, or 3 were assigned to each of these variables due to increasing abnormality, and the score calculated as the sum of the five variables for each patient. A Child-Pugh score less than 7 was considered class A, from 7 to 9 class B, while and any score greater than 9 was class C.

One experienced endoscopist assessed varices using a four-grade classification. Varices not arising from surrounding mucosa were recognized as grade I, those smaller than 5 mm fulfilling less than one-third of the oesophageal lumen were recognized as grade II, grade III were varices larger than 5 mm fulfilling more than one-third of the oesophageal lumen, while grade IV varices occupied more than two-thirds of the oesophageal lumen. The presence of a mosaic pattern and speckled red spots of the mucosa was termed portal hypertensive gastropathy.

The control group consisted of 218 healthy blood donors.

Blood samples were taken from the patients, sera separated by centrifugation and stored at -80°C until analysed.

Pi phenotype was determined using the isoelectric focusing (pH range 4.2–4.9) method described by Kishimoto et al. (8).

Biochemical parameters and liver enzymes were determined using routine laboratory methods.

### Statistical analysis

The chi-square test was used to assess if the control and study groups were in Hardy-Weinberg equilibrium for the AAT gene. Differences in frequency of AAT phenotypes and alleles as well as Pi M homozygosity and Pi M heterozygosity between study and control groups were tested using a chi-square association test (2 × 2 contingency table). If *n* < 5 we used the Fisher exact test.

Population characteristics were analysed using descriptive statistics and data shown as mean (interquartile range). Differences between men and women were evaluated by Mann-Whitney U-test. *P*-values of <0.05 were considered as significant. For statistical analysis, we used STATISTICA 6.0^®^ software.

## Results

Clinical and demographic data of the study group can be seen in Table I. Significantly higher incidences of smoking and alcohol consumption, but also positive autoimmune markers, varices grade III, and portal gastropathy were observed in male compared to female patients.

**Table I.** Clinical and demographic data of investigated adults with liver disease.

	All (*n* = 70)	Men (*n* = 46)	Women (*n* = 24)	*P*^a^
Age, year	54.51 ± 11.8	53.73 ± 11.59	56.04 ± 12.23	0.895
Smoking, %	63.4	82	25	<0.01
Liver cirrhosis, %	90	91	88	0.906
Hepatocellular carcinoma, %	10	9	12	0.652
Hepatitis B virus (HBV), %	2.8	4.2	0	0.910
Hepatitis C virus (HCV), %	25.3	25.5	25.4	0.999
Autoimmune markers, %	21.1	54.2	4.3	<0.01
Alcohol consumption, %	53.5	72.3	16.7	<0.01
Varices, %	57.7	68.0	37.5	<0.01
Stage:				
I	39.0	34.4	55.5	0.815
I	34.1	34.4	33.3	0.965
III	17.1	21.9	0	<0.05
IV	9.7	9.4	11.1	0.814
Portal gastropathy, %	33.8	44.7	12.5	<0.01
Child-Pugh score, %				
A	45.6	40.4	54.2	0.925
B	20.1	29.7	29.2	0.914
C	24.3	27.6	16.7	0.897

^a^Difference between men and women.

The distribution of AAT phenotypes and AAT alleles in the study group and controls are shown in Table II. No deviation from Hardy-Weinberg equilibrium was detected in controls (chi-square = 3.56; df 11; *P* = 0.981), while in the study group deviation from Hardy-Weinberg equilibrium (chi-square = 34.77, df 11, *P* = 0.000) was caused by an excess of Pi ZZ homozygotes (*f* = 0.0143 and *f* = 0.0005, respectively, *P* = 0.000).

**Table II.** Distribution of alpha-1-antitrypsin phenotypes and gene frequencies in 218 healthy individuals and 70 adults with liver disease.

	Observed/expected relative frequency		*P*^a^			Relative frequencies	
Phenotype	Patients	Control	Patients	Control	Allele	Patients	Control
M1	0.5429/0.5205	0.4083/0.3978	0.793	0.807	M1	0.7214	0.6307
M2	0.0857/0.0490	0.0413/0.0426	0.162	0.924	M2	0.2214	0.2064
M3	0.0000/0.0013	0.0092/0.0177	0.765	0.344	M3	0.0357	0.1330
M1M2	0.2714/0.3195	0.2431/0.2604	0.476	0.617	Z	0.0214	0.0183
M1M3	0.0714/0.0515	0.1697/0.1678	0.463	0.945	S	0.0000	0.0115
M2M3	0.0000/0.0158	0.0688/0.0549	0.292	0.381			
M1Z	0.0143/0.0309	0.0229/0.0231	0.428	0.983			
M2Z	0.0000/0.0095	0.0092/0.0076	0.415	0.786			
M3Z	0.0000/0.0015	0.0046/0.0049	0.743	0.950			
ZZ	0.0143/0.0005	0.0000/0.0000	0.000	1.000			
M1S	0.0000/0.0000	0.0092/0.0145	1.000	0.515			
M2S	0.0000/0.0000	0.0092/0.0047	1.000	0.340			
M3S	0.0000/0.0000	0.0046/0.0031	1.000	0.681			

^a^Differences between observed and expected relative frequency of AAT phenotypes.

Differences in the distributions of Pi phenotypes and gene frequencies between patients and controls are seen in Table III. In our study group Pi M1 homozygotes were more frequent than in controls (*f* = 0.5429 and *f* = 0.4083 respectively, *P* = 0.048). In the study group both Pi M1M3 and Pi M2M3 heterozygotes were less frequent than in the control group (M1M3: *f* = 0.0714 and *f* = 0.1697 respectively, *P* = 0.035; fM2M3: *f* = 0.0000 and *f* = 0.0688, respectively, *P* = 0.029).

**Table III.** Comparison of frequencies of alpha-1-antitrypsin phenotype and alleles between adults with liver disease and control.

	Relative frequency		
Phenotype	Patients	Control	*P*^a^
M1	0.5429	0.4083	0.048
M2	0.0857	0.0413	0.120
M3	0.0000	0.0092	0.471
M1M2	0.2714	0.2431	0.667
M1M3	0.0714	0.1697	0.035
M2M3	0.0000	0.0688	0.029
M1Z	0.0143	0.0229	0.682
M2Z	0.0000	0.0092	0.632
M3Z	0.0000	0.0046	0.795
ZZ	0.0143	0.0000	0.204
M1S	0.0000	0.0092	0.632
M2S	0.0000	0.0092	0.632
M3S	0.0000	0.0046	0.755
Allele			*P*^b^
M1	0.7214	0.6307	0.050
M2	0.2214	0.2064	0.686
M3	0.0357	0.1330	0.001
Z	0.0214	0.0183	0.694
S	0.0000	0.0115	0.259

^a^*P* differences in frequencies of AAT phenotypes between patients and control. ^b^*P* differences in frequencies of AAT alleles between patients and control.

Patients harboured the M1 allele more often than did controls (*f* = 0.7214 and 0.6307, respectively, *P* = 0.05). On the other hand, the M3 allele was less frequent in patients than in controls (*f* = 0.0357 and 0.1330, respectively, *P* = 0.01),

Our results revealed differences in distribution between Pi M homozygotes (Pi M1M1, Pi M2M2, and Pi M3M3) and Pi M heterozygotes (Pi M1M2, Pi M1M3, and Pi M2M3), with a  higher prevalence of Pi M homozygosity in the study group compared to controls (63% and 46%, respectively, *P* = 0.025).

Our study did not reveal differences in liver enzyme activity, severity of liver disease, and risk factors for liver disease related to the presence of the Z allele in our study group (Table IV).

**Table IV:** Characteristics of adults with liver disease according to Z allele.

	Non-carriers of Z allele (*n* = 68)	Carriers of Z allele (*n* = 2)	*P*^a^
Liver cirrhosis, %	90	50	0.193
Hepatocellular carcinoma, %	10	50	0.193
AST^b^, UL^-1^	97.33 (37–88)	35.50 (37–40)	0.266
ALT^c^, UL^-1^	72.63 (32–66.5)	37 (21–53)	0.332
AP^d^, UL^-1^	165.36 (76–136)	212.5 (84–341)	0.543
GGT^e^, UL^-1^	165.51 (32.5–245.5)	286.00 (168–404)	0.217
Hepatitis B virus (HBV), %	4.4	0	0.915
Hepatitis C virus (HCV), %	25	50	0.405
Autoimmune markers, %	21	50	0.385
Alcohol consumption, %	54	50	0.708
Varices, %	58	0	0.180
Portal gastropathy, %	40	0	0.374
Child-Pugh score, %			
A	43	100	0.205
B	29	0	0.507
C	25	0	0.571

^a^Differences between non-carriers and carriers of the Z allele; ^b^aspartate aminotransferase; ^c^alanine aminotransferase; ^d^alkaline phosphatase; ^e^gamma glutamyl transferase.

## Discussion

Of all AAT alleles and variants, only AAT variants associated with low AAT plasma levels termed alpha-1-antitrypsin deficiency (AATD) are of clinical importance. Risk of AATD-related liver disease in childhood has been previously well documented. Results of a large neonatal screening study by Sveger (9) and other epidemiological studies (10–14) demonstrated that Pi ZZ-carrying infants have clinical signs of neonatal cholestasis and other clinical symptoms of liver disease without jaundice. Although the majority of children with AATD (83%) are clinically healthy throughout childhood, it is estimated that 14%–46% of all liver transplantation in paediatric patients is due to liver failure caused by AATD (15,16).

In adults the association of AATD and liver disease is less clear than in children. In a recent review Kok et al. (17) stated that adult Pi ZZ carriers had a higher risk of developing end stage liver disease, cirrhosis, and hepatocellular carcinoma (HCC). Heterozygous AATD is an important co-factor in the aetiology of chronic liver disease, and it can modify the course of hepatitis C, end stage liver disease, cirrhosis, and hepatocellular carcinoma. Therefore clarifying the association between heterozygous AATD and liver disease in the adult population is of clinical importance.

In our study 90% of patients had liver cirrhosis of different aetiology, and 10% were diagnosed with hepatocellular carcinoma.

Patients with liver disease had a higher frequency of Pi ZZ homozygosity than expected, which caused deviation from Hardy-Weinberg equilibrium and supported the previously proposed association between Pi ZZ genotype and severe liver disease. A large retrospective study (2) demonstrated an increased risk of cirrhosis and liver cancer in Pi ZZ individuals. In addition, Elzouki and Eriksson (18) reported that male, Pi ZZ homozygous individuals have a higher risk of developing cirrhosis and HCC, independently of hepatitis B or C infection. Our results in the study group support these findings.

However, we failed to demonstrate a different frequency of Pi ZZ homozygosity between patients with liver disease and healthy individuals, probably due to a relatively small number of patients in the study group.

The possible role of the Z allele in the pathogenesis of chronic liver disease in heterozygous AATD patients is still controversial. Some studies provide evidence of an association between the heterozygous Z allele alpha-1-antitrypsin phenotype and end stage liver disease of different aetiology (19–22), but other studies (including ours) failed to identify an association between heterozygous MZ alpha-1antitrypsin deficiency and cirrhosis (23–27). We were not able to show differences in Pi MZ frequency between patients and healthy controls. The study by Rakela et al. (28) showed that liver disease became symptomatic at the age of 58 years in Pi ZZ, 66 years in Pi SZ, and 73 years in Pi MZ carriers. The mean age of our Pi MZ carriers was 54 ± 11 years which could explain why we could not confirm an association between Pi MZ phenotype and liver disease.

We could not identify differences in the activity of liver enzymes and the degree of liver failure (Child-Pugh score, varices, and portal hypertensive gastropathy) in patients relative to the presence of Z allele.

According to our results, there is no difference in aetiology of cirrhosis related to the presence of Z allele carriers, implying that adult Z allele carriers were not more sensitive to any of the known risk factors for liver disease. Results from our study partially agree with the data from the literature. Vogel et al. (29) documented that hepatitis C virus (HCV) infection is an exogenous risk factor which is required for the development of chronic liver disease in Z allele carriers (homozygotes and heterozygotes), but hepatitis B virus (HBV), alcohol abuse, and autoimmunity were not risk factors. A recent study (19) showed that AATD might be a risk factor for infected HBV individuals progressing from the carrier stage to chronic and cirrhotic stages. Bowlus et al. (30) reported that male gender and obesity, but not alcohol or viral hepatitis, predispose to advanced liver disease in adults with AATD.

Surprisingly, we report a significant difference in distribution of Pi M subtypes between the patient and control groups. This result is unexpected, because Pi M subtypes are not AAT-deficient and not related to clinical diseases. In our study Pi M1 homozygosity was more frequent in patients than in the controls, and both Pi M1M3 and Pi M2M3 heterozygosity was less frequent in patients than in controls. Further analysis of AAT allele frequency revealed that the M1 allele was more frequent and the M3 allele less frequent in patients than in controls. In most populations, the M3 allele is less frequent compared to the M1 and M2 alleles, and the relatively small number of patients included in our study could explain the reduced frequency of M3 allele. It is interesting that more M homozygosity was found in patients than in the control group, which could be explained by the advantages of Pi M heterozygosity in a healthy individual. In addition, these findings suggest that M homozygotes could be associated with liver disease via some mechanism different from Z allele-induced hepatocyte damage through accumulation of AAT polymers, but this finding needs to be confirmed in larger studies.
